# Prevalence of psoriatic arthritis in Italy: insights from the multicentric MAPSI study

**DOI:** 10.3389/fmed.2024.1484988

**Published:** 2025-01-06

**Authors:** Teodora Serban, Giuseppina Tramontano, Monica Pendolino, Dario Roccatello, Oscar Massimiliano Epis, Florienzo Iannone, Ilenia De Andres, Marta Favero, Nicoletta Romeo, Guido Rovera, Leonardo Santo, Enrico Tirri, Alberto Bergamini, Roberta Foti, Carlotta Schenone, Gerolamo Bianchi

**Affiliations:** ^1^S.C. Reumatologia, ASL3 Genovese, Genoa, Italy; ^2^University Center of Excellence on Nephrologic, Rheumatologic and Rare Diseases (ERK-net, ERN-Reconnect and RITA-ERN Member), ASL Città di Torino and Department of Clinical and Biological Sciences, University of Turin, Turin, Italy; ^3^Division of Rheumatology, Multispecialist Medical Department, Grande Ospedale Metropolitano Niguarda, Milan, Italy; ^4^Rheumatology Unit, DiMePRE-J, University of Bari, Bari, Italy; ^5^Rheumatology Unit, Azienda Ospedaliera di Rilievo Nazionale ed Alta Specializzazione “Garibaldi”, Catania, Italy; ^6^Internal Medicine 1, Ca’ Foncello University Hospital, AULSS2, Treviso, Italy; ^7^Azienda Ospedaliera S Croce e Carle, Cuneo, Italy; ^8^Nuclear Medicine Division, Department of Medical Sciences, University of Turin, Turin, Italy; ^9^Rheumatology Unit, “Mons. Dimiccoli” Hospital, Barletta, Italy; ^10^UOSD di Reumatologia, Ospedale San Giovanni Bosco, Napoli, Italy; ^11^Rheumatology, Department of Systems Medicine, University of Rome Tor Vergata, Rome, Italy; ^12^Division of Rheumatology, A.O.U. “Policlinico-San Marco”, Catania, Italy

**Keywords:** psoriatic arthritis, psoriasis, gender differences, prevalence, real world data

## Abstract

**Introduction:**

Psoriatic arthritis (PsA) is a chronic inflammatory arthropathy associated with cutaneous psoriasis (PsO), first defined by Moll and Wright. Initially perceived as relatively benign, PsA is now recognized for its chronic, progressive, and destructive nature, significantly impacting patients' quality of life, similar to Rheumatoid Arthritis (RA). Globally, PsA represents about 20% of cases in early arthritis clinics, posing diagnostic and management challenges. Early diagnosis is crucial to prevent long-term disability and prevent comorbidities. While classification criteria for PsA are widely accepted, the lack of specific diagnostic criteria may delay diagnosis, with many patients experiencing delays of over a year before receiving an accurate diagnosis. For this reason, the exact prevalence of PsA remains uncertain.

**Methods:**

The MAnagement of PSoriatic Arthritis in Italy (MAPSI) project is a multicenter observational study aimed to investigate the prevalence of PsA and characteristics in an Italian cohort. This study included 454 patients diagnosed or confirmed by a rheumatologist across 25 Rheumatology Units in Italy. Data were collected on demographics, clinical features, and treatment histories. In our cohort, distinct gender differences were observed in PsA phenotype and disease impact.

**Results:**

Men had a higher prevalence of axial involvement and were more likely to be current or former smokers, while women showed greater rates of enthesitis and reported higher perceived disease activity. Additionally, women had a higher prevalence of psychological comorbidities, whereas men had more severe skin involvement; laboratory tests were often unhelpful in diagnosing PsA, with elevated inflammatory markers in only about half of the cases.

**Discussion:**

These findings highlight the need for gender-sensitive approaches in the management of PsA. Our findings highlight the importance of comprehensive patient evaluation, including extramuscoloskeletal manifestation with a gender-sensitive approaches, focusing on a prompt diagnosis to prevent systemic comorbidities. Real-world data, such as those provided by the MAPSI study, are essential for understanding the burden of PsA and developing effective management strategies tailored to patient characteristics.

## 1 Introduction

Psoriatic arthritis (PsA) was initially defined by Moll and Wright as a chronic inflammatory arthritis usually seronegative associated with cutaneous psoriasis (PsO) (1–5). Although initially considered a relatively benign disease, nowadays is well known its chronic, progressive, and destructive nature, impacting quality of life of patients similarly to rheumatoid arthritis (RA) (6, 7).

Worldwide, PsA represents around 20% of cases referred to early arthritis clinics, constituting a real challenge from the point of view of diagnosis and management (8–10). Early diagnosis is essential to prevent long-term functional disability and to ensure optimal management of arthritis and its key comorbidities.

Although classification criteria for PsA are widely accepted, the lack of specific diagnostic criteria can lead to delayed diagnoses, with many patients experiencing diagnostic delays exceeding 1 year (11–13). This contributes to uncertain prevalence of PsA (14–16).

In addition to assessing the different clinical domains of PsA—polyarticular, oligoarticular, enthesitic, dactylitic, axial, and onichophaty—it is crucial to fully evaluate the spectrum of extra-articular manifestations, such as inflammatory bowel disease (IBD) and uveitis, along with any comorbidities the patient may experience (2, 17, 18). A more comprehensive characterization of the patient will facilitate the personalized therapeutic strategy, tailored to address the specific needs of each patient.

The MAnagement of PSoriatic arthritis in Italy (MAPSI) project is a multicentric observational study designed to investigate the prevalence, focusing on gender distribution among the different clinical patterns of presentation of an Italian cohort of PsA patients.

## 2 Materials and methods

### 2.1 Study design

The MAPSI project is a non-profit, multicenter, cross-sectional, and observational study, explicitly designed as non-retrospective, designed to investigate the prevalence of PsA in Italy. The study was conducted across twenty-five Rheumatology Units in Italy, recruiting patients from both hospital-based and outpatient clinics; see the Supplementary material for information on enrollment centers and patient numbers per center. Data were collected concurrently with patient evaluations, beginning with an initial baseline visit (T0) and, where applicable, a follow-up visit to monitor clinical progression.

### 2.2 Study population

Patients with a diagnosis of PsA, either formulated or confirmed by a rheumatologist, were enrolled in the study. Recruitment from a variety of clinical settings aimed to achieve a representative sample of the Italian PsA population. Inclusion criteria mandated a confirmed PsA diagnosis by a rheumatologist, ensuring that all cases met standard clinical practice guidelines.

### 2.3 Ethical considerations

The study protocol was approved by the Ethics Committee of each participating center, in compliance with ethical guidelines for research. All patients provided written informed consent before enrollment, confirming their willingness to participate in the study and allowing the use of their data for research purposes.

### 2.4 Assessment

At T0 treating physicians collected a comprehensive set of demographic, clinical, therapeutic, clinimetric indices and laboratory data for each patient. Baseline demographic data included gender, age, body mass index (BMI), and smoking status.

Disease-specific data were collected through an in-depth anamnesis to ensure accuracy and detail. All data were systematically recorded using case report forms (CRFs) to standardize data collection across centers. We documented data concerning the onset of psoriatic arthritis, including age at disease onset, disease duration at the time of diagnosis, and symptom duration prior to diagnosis. Clinical presentation was documented based on physician-assessed joint counts for peripheral arthritis, dactylitis, nail psoriasis, skin psoriasis and enthesitis domain. For axial involvement, magnetic resonance imaging (MRI) assessments were performed to provide potential involvement. Furthermore, clinical assessments included the number of tender joints (TJC) and swollen joints (SJC), patient-reported visual analog scale (VAS) scores for general health, pain, and disease activity, as well as the physician's VAS score for disease activity (MGA). Laboratory and clinimetric indices were also recorded, including C-reactive protein (CRP, mg/dl), Disease Activity in Psoriatic Arthritis Score-28 (DAPSA28)-CRP score, Health Assessment Questionnaire (HAQ), Disease Activity in Psoriatic Arthritis (DAPSA), Bath Ankylosing Spondylitis Disease Activity Index (BASDAI), Psoriasis Area Severity Index (PASI), Nail Psoriasis Severity Index (NAPSI), Maastricht Ankylosing Spondylitis Enthesis Score (MASES), and Leeds Enthesitis Index (LEI). Moreover, additional data regarding comorbidities, and pharmacological therapy were also documented.

### 2.5 Outcome

The primary outcome of this study was to investigate the prevalence characteristics of PsA in the Italian population, with a specific focus on gender distribution and clinical domain of disease presentation. The study aimed to assess and compare the prevalence of various PsA phenotypes, between gender groups and to enhance understanding of PsA presentation in Italy and support targeted management strategies by highlighting potential differences in disease characteristics between males and females.

### 2.6 Statistical analysis

Continuous variables were reported as mean ± standard deviation (SD) or median [interquartile range, (IQR)] depending on distribution. Categorical variables were reported as numbers and percentages. Differences between groups were assessed using the ANOVA or the independent *t*-test for continuous variables or by Pearson's χ^2^ test for dichotomous variables. Probability (p) values < 0.05 were considered statistically significant. All analyses were performed using SPSS software 22.0 (Chicago, SPSS, Inc.).

### 2.7 Statistical analysis

Continuous variables were reported as mean ± standard deviation (SD) or median [interquartile range, (IQR)] depending on distribution. Categorical variables were reported as numbers and percentages. Differences between groups were assessed using the ANOVA or the independent *t*-test for continuous variables or by Pearson's χ^2^ test for dichotomous variables. Probability (p) values < 0.05 were considered statistically significant. All analyses were performed using SPSS software 22.0 (Chicago, SPSS, Inc.).

## 3 Results

A total of 454 patients with PsA were enrolled in the MAPSI study; the mean age at enrollment was 55.45 ± 11.52 years, reflecting the age range typically associated with PsA onset and progression. The main demographic, anthropometric and clinical characteristics of patients are summarized in Table 1.

**Table 1:** Demographic and disease characteristics at baseline.

**A: Demographic data**	
Number of patients	454
Age, year (mean; SD)	55.4;11.5
Female (*n*; %)	239; 52.6%
Weight, Kg (mean; SD)	77; 16.7
BMI	27.3; 5.3
Active smokers (*n*; %)	65; 14.3%
Joint symptoms before diagnosis, years [mean; (IQR)]	9; (4–18)
Age at PsA diagnosis, month (mean; SD)	46.7; 12.3
Disease duration, months [median; (IQR)]	7; (3–12)
**B: Clinimetric indices**	
Pain VAS, mm (mean; SD)	40.9; 29.1
PGA, mm (mean; SD)	40.9; 28.7
MGA, mm (mean; SD)	28.9; 26.4
GH, mm (mean; SD)	48.3; 27.4
CRP, mg/dl [median;(IQR)]	0.3; (0.1–0.6)
TJC [median; (IQR)]	1; (0–5)
SJC [median; (IQR)]	0 (0−1)
DAS28 (mean; SD)	3.0; 1.21
HAQ (mean; SD)	0.7; 0.69
BASDAI (mean; SD)	3.57; 2.91
LEI [median; (IQR)]	0; (0–1)
MASES [median; (IQR)]	0; (0–0)
TDS [median; (IQR)]	0; (0–0)
DAPSA [median; (IQR)]	11.5; (4–24)
PsAID9 (mean; SD)	4.0; 2.85
PASI [median; (IQR)]	0 (0–0.9)
NAPSI [median; (IQR)]	0 (0–0)
**C: Treatment**	
NSAIDs (*n*; %)	150; 33
Glucocorticoids (*n*; %)	102; 22.5
cDMARDs (*n*; %)	238; 52.4
b-tDMARDs (*n*; %)	284; 62.6
Other (*n*; %)	57; 12.6

n, number; PsA, psoriatic arthritis; VAS, visual analog scale; PGA/patient global assessment; MGA, medical global assessment; GH, general health; CRP, C reactive protein; TJC, tender joint count; SJC, swollen joint count; DAS28, disease activity score (28 joints); HAQ, health assessment questionnaire; BASDAI, Bath Ankylosing Spondylitis Disease Activity Index; LEI, Leeds Enthesitis Index; MASES, Maastrich Ankylosing Spondylitis Enthesitis Score; TDS, Tender Dactilitys Score; DAPSA, Disease Activity in PSoriatic Arthritis; PsAID9, Psoriatic Arthritis Impact of Disease; PASI, Psoriasis Area and Severity Score; NAPSI, Nail Psoriasis Severity Index; NSAID, Nonsteroidal Anti-Inflammatory Drugs; cDMARDs, Conventional Disease-Modifying Antirheumatic Drugs; b-tDMARDs, Biologic and Target Disease-Modifying Antirheumatic Drugs.

The median duration of symptoms prior to diagnosis was 9 months (IQR: 3–14 months). Despite this relatively short median delay, only 72.6% of patients received a diagnosis within the 1^st^ year after symptom onset, the potential diagnostic delays could impact early treatment interventions.

Most patients (80.8%) were diagnosed by a rheumatologist, and a substantial majority (79.1%) met the Classification Criteria for Psoriatic Arthritis (CASPAR) at the time of diagnosis. However, data on the number of joints affected at disease onset and the exact time from symptom onset to diagnosis were not available for over half of the patients (57% and 50.2%, respectively), which may limit the study's precision in assessing initial disease presentation.

Significantly more men (18.9%) were current or former smokers compared to women (3.6%) (*p* = 0.004). This prevalence difference in smoking history may have implications for PsA disease activities and comorbidity profiles, which may need further longitudinal analysis. The majority of patients did not show statistically significant correlations with CRP levels, confirming findings reported in the literature (19).

### 3.1 Body mass index and gender distribution

The mean BMI in this cohort was notably similar between genders, though slightly higher in males (27.57 for males vs. 27.05 for females). However, obesity (defined as BMI ≥ 30) was observed in 31.46% of females compared to 24.32% of males, this distribution suggests potential gender-based differences in weight-related health impacts, possibly contributing to differential disease experiences.

### 3.2 Prevalence of comorbidities and gender differences

Cardiovascular and metabolic comorbidities were common in this cohort, with 49.77% of males and 39.75% of females affected by at least one cardiometabolic condition (e.g., hypertension, diabetes, or dyslipidemia). Conversely, psychological comorbidities, including depression and anxiety, were more frequently reported among females (10.46%) than males (5.58%). These findings indicate a higher prevalence of psychological impacts in women with PsA, which may contribute to perceived disease burden. Inflammatory comorbidities, including inflammatory bowel disease (IBD) and uveitis, had similar rates between genders, with a prevalence of approximately 3% in both groups.

### 3.3 PsA phenotype prevalence

**Figure 1:**
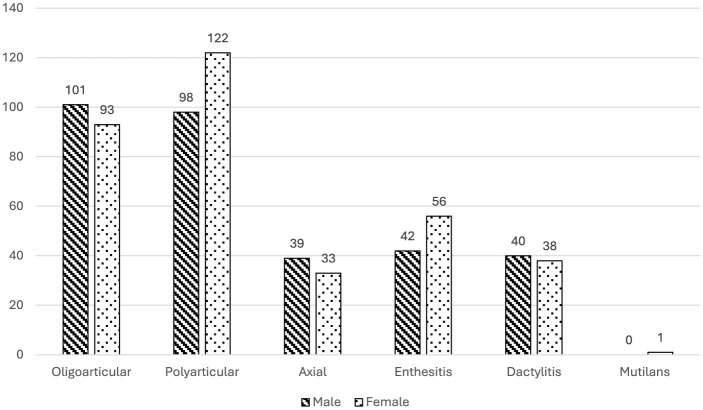
Gender Differences in PsA Phenotype Prevalence with Patient Counts: This bar chart illustrates the prevalence of various PsA phenotypes among male and female patients in the MAPSI study cohort at baseline. The phenotypes include oligoarticular, polyarticular, axial involvement, enthesitis, dactylitis, and mutilans. Striped bars represent male patients, and dotted bars represent female patients. Significant gender differences were observed in axial involvement and enthesitis prevalence, with higher rates of axial involvement in males (*p* = 0.012) and enthesitis in females (*p* = 0.044).

In terms of phenotype distribution: peripheral arthritis was the most prevalent form, observed in 91.2% of patients, with 42.7% showing an oligoarticular pattern and 48.5% a polyarticular pattern; enthesitis was present in 21.6% of patients; dactylitis was noted in 17.2%; axial involvement was observed in 15.9% and mutilans form was extremely rare in our cohort, occurring in only one patient (0.2%) (Figure 1). Most patients (62.6%) exhibited only one PsA phenotype at the time of assessment, while a smaller proportion (8.6%) presented with three distinct phenotypes, suggesting considerable heterogeneity in clinical presentation within this population.

### 3.4 Gender differences in phenotype prevalence

Distinct gender-related differences were observed across phenotypes; while the overall prevalence of PsA is balanced between genders in our cohort (*p* = 0.385), distinct gender-based differences emerge when examining specific phenotypes. Men are significantly more likely to exhibit axial involvement (*p* = 0.012), whereas women show a higher prevalence of enthesitis (*p* = 0.044) (Figure 1).

**Figure 2:**
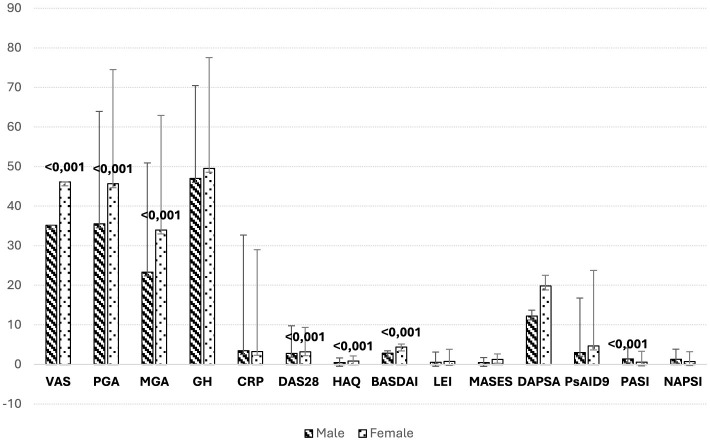
Comparison of clinimetric indices by gender. Mean scores for various clinimetric indices in male and female PsA patients from the MAPSI cohort, with error bars representing the standard deviation. Women generally report higher disease activity, as indicated by elevated scores in VAS, PGA, DAS28, and HAQ. These findings align with the study's results, suggesting a higher perceived burden of disease among female patients. Conversely, men exhibit higher scores on PASI, reflecting more severe skin involvement. This gender-specific pattern in disease activity and severity highlights the importance of tailored management strategies in PsA, considering differences in both perceived and clinically assessed disease domains. VAS, visual analog scale; PGA/patient global assessment; MGA, medical global assessment; GH, general health; CRP, C reactive protein; TJC, tender joint count; SJC, swollen joint count; DAS28, disease activity score (28 joints); HAQ, health assessment questionnaire; BASDAI, Bath Ankylosing Spondylitis Disease Activity Index; LEI, Leeds Enthesitis Index; MASES, Maastrich Ankylosing Spondylitis Enthesitis Score; TDS, Tender Dactilitys Score; DAPSA, Disease Activity in PSoriatic Arthritis; PsAID9, Psoriatic Arthritis Impact of Disease; PASI, Psoriasis Area and Severity Score; NAPSI, Nail Psoriasis Severity Index.

Additionally, women reported higher scores for disease activity, reflected in the BASDAI, VAS, TJC, DAS28, and HAQ scores (all *p* < 0.05), indicating a higher burden of perceived disease activity. In contrast, men had significantly higher PASI scores (*p* < 0.001), suggesting more severe skin involvement. Further analysis of disease activity indices demonstrated a higher prevalence of elevated MASES scores among women (*p* = 0.022), specifically indicating more frequent entheseal involvement, although LEI scores did not show a significant gender difference (*p* > 0.05) (Figure 2).

These findings suggest that, despite similar overall prevalence, certain clinical manifestations of PsA vary by gender, underscoring the importance of gender-sensitive approaches in the management and assessment of PsA.

### 3.5 Summary of prevalence findings

Overall, this cross-sectional analysis indicates that peripheral arthritis is the most prevalent PsA phenotype in this Italian cohort, with notable gender-specific variations in axial and enthesitic involvement. Additionally, the observed delays in diagnosis for a significant proportion of patients underscore the need for improved awareness and screening practices in PsA. The identified prevalence of smoking among male patients and the increased skin severity in males highlight potential areas for targeted interventions.

The main demographic, anthropometric and clinical characteristics of patients are summarized in Table 1. Significantly more men (86 patients [18, 94%]) were current or past smokers in confront with women.

## 4 Discussion

This study provides a detailed overview of the clinical and demographic characteristics of PsA patients in an Italian cohort, highlighting important gender-based differences. The balanced gender distribution aligns with known PsA demographics, though females tended to report higher disease burden.

A median diagnostic delay of 9 months, with over a quarter of patients waiting more than a year, suggests potential impacts on long-term outcomes (20, 21). This delay emphasizes the need for improved PsA screening, as early diagnosis is crucial for effective management (22). The high rate of diagnoses by rheumatologists and the large proportion meeting CASPAR criteria (79.1%) underline the importance of specialized assessment, although these criteria may have limited sensitivity for early PsA.

Notable gender differences emerged: males had higher smoking prevalence and cardiometabolic comorbidities, while females showed more obesity and psychological comorbidities (23, 24). These distinctions highlight the need for tailored approaches addressing specific comorbidities in PsA management (25, 26).

The predominance of peripheral arthritis, seen in 91.2% of patients, with significant gender variations in specific phenotypes (axial involvement in males, enthesitis in females), reinforces the heterogeneous nature of PsA (27, 28). Women reported higher disease activity scores, while men had greater skin involvement, suggesting gender-specific disease experiences.

In summary, this study highlights significant gender differences and diagnostic delays in PsA. These findings support the need for gender-sensitive approaches in PsA management, timely diagnosis, and tailored interventions that consider distinct comorbidities and disease expressions across genders.

## 5 Conclusion

In summary, while the exact prevalence of PsA has been less extensively studied compared to many other rheumatic diseases, our study provides valuable real-world data on Italian patients affected by PsA, offering key insights into the patient characteristics that can inform effective treatment strategies. We found that, although the prevalence of PsA is balanced between genders, significant gender-specific differences exist in clinical manifestation. These findings underscore the importance of a gender-sensitive approach to the management and assessment of PsA, highlighting the need for tailored interventions to address the unique clinical presentations and comorbidity profiles in each gender (29, 30). Further longitudinal research is essential to understand how these gender-based differences impact long-term disease progression and treatment outcomes.

### 5.1 Limitation of the study

This study provides valuable insights into the prevalence and characteristics of PsA phenotypes, comorbidities, and gender differences within an Italian cohort. However, several limitations should be considered when interpreting the findings. First, the absence of a non-PsA comparison group or general population data limits the ability to contextualize observed prevalence rates and demographic patterns beyond the PsA population studies. Additionally, the cross-sectional design restricts causal inferences and prevents tracking the evolution of disease phenotypes, comorbidities, and severity indicators over time; longitudinal data would be beneficial to assess the progression and long-term outcomes associated with these factors in PsA. Furthermore, the study cohort was sourced from a single geographic region, consequently affect the applicability of these results to different populations. Finally, missing data on important factors—like the time from symptom onset to diagnosis and the number of affected joints at disease start—was noted in over half of the patients. This limits our ability to fully understand early disease presentation and how delays in diagnosis might affect outcomes. Future research could address these gaps by using long-term studies, comparing with broader populations, and collecting more complete data to better understand PsA progression and management.

## Data Availability

The raw data supporting the conclusions of this article will be made available by the authors, without undue reservation.
