# Supplementary figures and images for: Prevalence of psoriatic arthritis in Italy: insights from the multicentric MAPSI study
Source: Front Med (Lausanne). 2025 Jan 6;11:1484988. doi: 10.3389/fmed.2024.1484988 (PMC11773365; doi:10.3389/fmed.2024.1484988)

**Supplementary files:** Enrollment rates across Italy

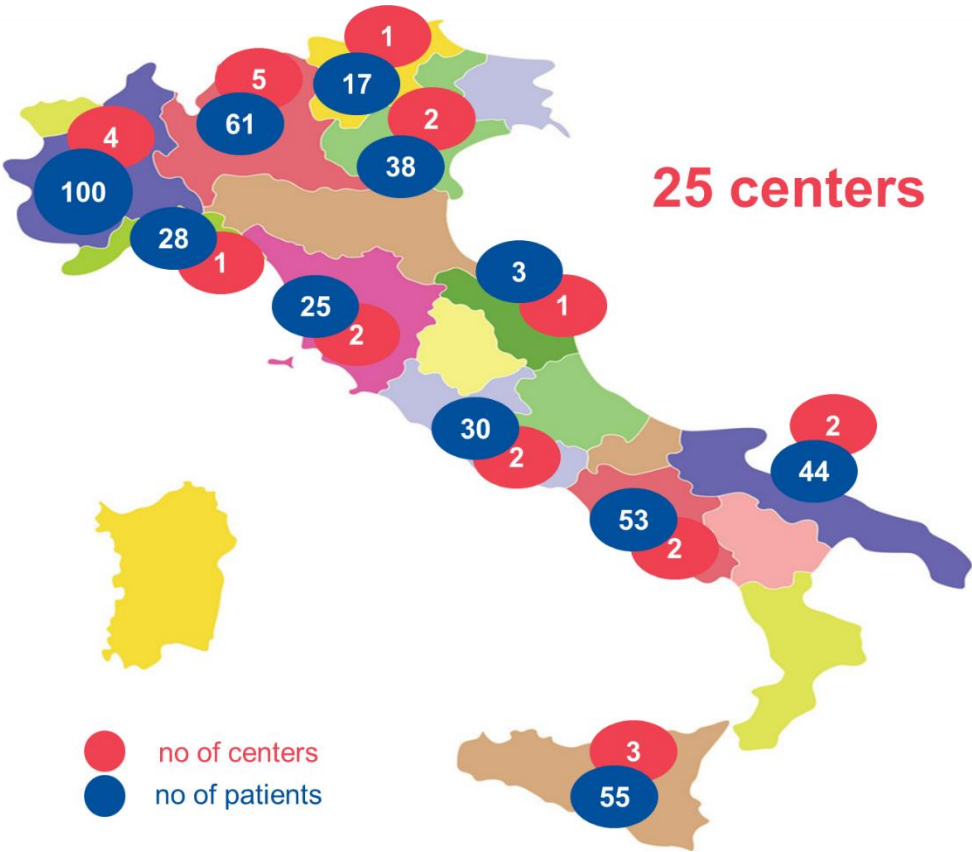

Supplement: Supplementary file 1 [file Image_1.pdf]
